# Supplementary material for: The Human Thyroid-Derived CI-huThyrEC Cell Line Expresses the Thyrotropin (TSH) Receptor and Thyroglobulin but Lacks Other Essential Characteristics of Thyroid Follicular Cells
Source: Biomolecules. 2025 Mar 5;15(3):375. doi: 10.3390/biom15030375 (PMC11940677; doi:10.3390/biom15030375)
Supplement: Supplementary file 1 [file biomolecules-15-00375-s001.zip › biomolecules-3362785-supplementary.pdf]

biomolecules

MDPI

Article

The Human Thyroid-Derived CI-huThyrEC Cell Line Expresses the Thyrotropin (TSH) Receptor and Thyroglobulin but Lacks Other Essential Characteristics of Thyroid Follicular Cells

Mathias Halbout [1,2] and Peter A. Kopp [1,2,*]

[1] Division of Endocrinology, Diabetes and Metabolism, University Hospital of Lausanne, University of Lausanne, Hôtel des Patients, Avenue de la Sallaz 08, CH-1011 Lausanne, Switzerland
[2] Faculty of Biology and Medicine, University of Lausanne, 1015 Lausanne, Switzerland
* Correspondence: peter.kopp@chuv.ch or peter.kopp@endocrine.ch; Tel.: +41-79-556-69-19

Abstract: ***Background:*** Thyroid hormone synthesis requires the normal function of thyroid follicular cells and adequate nutritional intake of iodine. For in vitro studies on thyroid cell pathophysiology, the immortalized FRTL5 rat thyroid cell line and a derivative thereof, the PCCL3 cell line, are widely used. However, a permanent human thyroid cell line is currently lacking. A recent report described a cell line obtained from human thyroid cells designated as Cl-huThyrEC. ***Methods***: Four clones of Cl-huThyrEC cells were ob-tained and cultured in the presence of thyroid stimulating hormone (TSH). The expression of key genes defining the thyroid follicular cell phenotype was determined by reverse-transcription PCR (RT-PCR) in FRTL5, PCCL3, and Cl-huThyrEC cells. The latter were cultured as monolayers and as organoids in Matrigel. Iodide uptake was measured and compared among the cell lines. ***Results:*** Gene expression analysis reveals that Cl-huThyrEC cells express the thyroid-restricted transcription factors (*PAX8*, *NKX2.1*, *FOXE1*), the TSH receptor (*TSHR*), and thyroglobulin (*TG*), but they do not express the sodium-iodide symporter (*NIS*), thyroid peroxidase (*TPO*), and pendrin (*SLC26A4*). In functional studies, Cl-huThyrEC cells are unable to concentrate iodide. ***Conclusions:*** De-spite the expression of certain key genes that are limited or restricted to thyroid follicular cells, Cl-huThyrEC cells lack some of the essential characteristics of thyroid follicular cells, in particular, *NIS*. Hence, their utility as a model system for thyroid follicular cells is lim-ited.

Keywords: thyroid; cell line; iodine; sodium iodide symporter; thyroid peroxidase

 https://doi.org/10.3390/biom15030375

1. Supplementary material

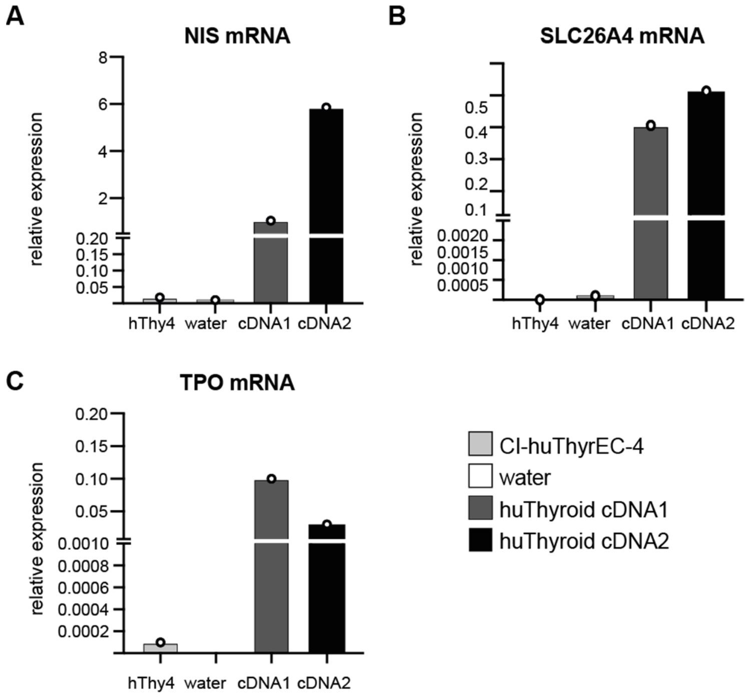

Figure S1. Relative expression of *NIS* (A), *SLC26A4* (B) and *TPO* (C), in the CI-huThyrEC clone four, water and two cDNAs samples from human thyroids. Results show the means of one experiment performed with technical duplicates.

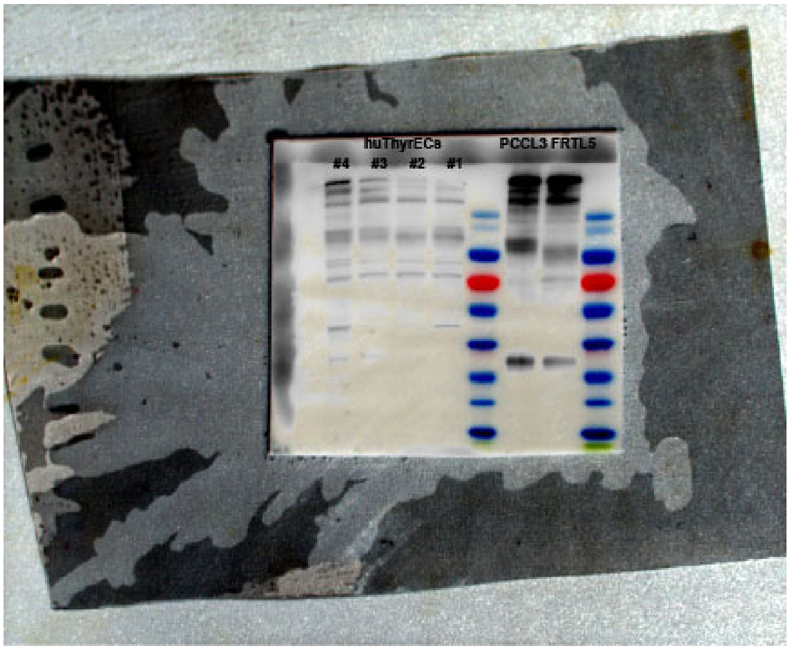

Figure S2.A

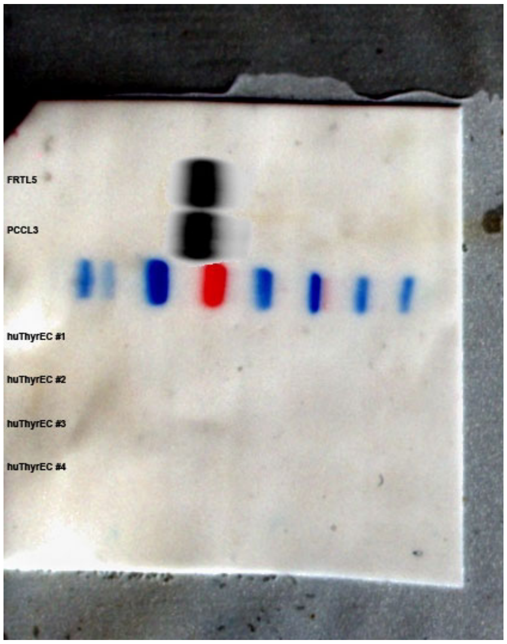

Figure S2.B

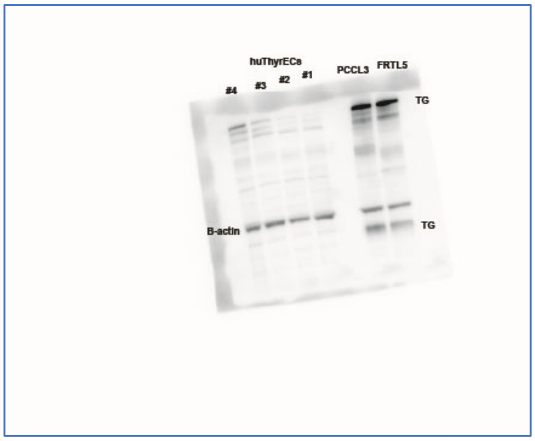

Figure S2.C

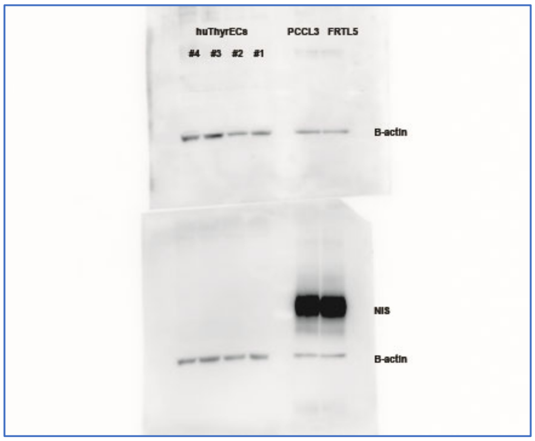

Figure S2.D

Figure S2. Original images of Western blot analysis of FRTL5, PCCL3, and the four clones from Cl-huThyrEC cell lines. Membranes were incubated with antibodies against thyroglobulin (TG, (S2.A)) and the sodium iodide symporter (NIS, (S2.B)) and merged with molecular weight markers (kilo Dalton, kDa). The membranes were then incubated with an antibody against B-actin (arrow) as a loading control (S2.C, S2.D).

Table S1. Table indicating the slope equation shown in Figure 6C and a linear regression to assess whether each slope is significantly deviated from 0.

	PCCL3	FRTL5	huThyr-4
**Equation**	Y = 25,40*X - 45,51	Y = 28,47*X - 37,61	Y = 0,3278*X - 2,233
**Is slope significantly non-zero?**			
F	55,72	110,5	1,61
DFn, DFd	1,13	1,13	1,13
P value	<0,0001	<0,0001	0,2268
Deviation from zero?	Significant	Significant	Not Significant

Table S2. Primer sequences used in this study.

Gene	forward primer	reverse primer	species
Tbp	tatcactcctgccacaccag	tgtgcacaccattttcccag	Rat
Beta-actin (Actb)	ccgcgagtacaaccttcttg	gacccatacccaccatcaca	Rat
Nis/Slc5a5	tggactttgaccctgatcct	acaagcagcggcatgtact	Rat
Pds/Slc26a4	cagtcccgattcctatagaa	gtaatttgcttccaagttgg	Rat
TBP	ctggtttgccaagaagaaag	gggtcagtccagtgccataa	Human
GAPDH	gtctcctctgacttcaacagcg	accaccctgttgctgtagccaa	Human
NIS/SLC5A5	ctctgctggtgctggacatctt	gaggtcttctacagtgactgcag	Human
PDS/SLC26A4	gacattcctcgtctgtggagac	gccagctagtaaaccgagatcc	Human
TG	ccagtggcttctcttcctgact	ccttggaggaagcggatggttt	Human
TPO	caccaggctttcttcagcccat	ggacagcacaaagagcctttcc	Human
TSHR	gagtttccttcacctcacacgg	ctgctctcattacacatcaaggac	Human
PAX8	tcaacctccctatggacagctg	gagcccattgatggagtaggtg	Human
NKX2.1	caggacaccatgaggaacagcg	gccatgttcttgctcacgtccc	Human
FOXE1	cacactcaacgactgcttcctc	caggaagctgccgctctcgaa	Human
